# From “Doing” to “Being”: How Agency and Identity Mediate the Associations of Support and Stress with Doctoral Competence in Chinese Universities

**DOI:** 10.3390/bs16101941

**Published:** 2026-10-19

**Authors:** Lingmei Huang, Fuqian Cui, Tengteng Zhuang

**Affiliations:** Institute of Higher Education, Faculty of Education, Beijing Normal University, Beijing 100875, China; hlm@bnu.edu.cn (L.H.); 202431010035@mail.bnu.edu.cn (F.C.)

**Keywords:** doctoral education, serial mediation, academic competence, agency, identity, job demands–resources

## Abstract

Doctoral students’ academic competence is not only a key indicator of doctoral training quality but also a core outcome of their professional socialization. Drawing on the Job Demands–Resources (JD–R) framework, this study developed a dual-pathway model to examine how supervisor support and research stress were associated with doctoral students’ academic competence. In particular, it investigated the sequential indirect associations involving doctoral student agency and research role identity. Based on 436 valid responses from doctoral students across disciplines and years of study at three comprehensive universities in Beijing, China, structural equation modeling was used to examine the hypothesized associations. Results were consistent with the proposed dual-pathway model. Supervisor support was associated with academic competence through agency and research role identity. The largest estimated specific indirect association was observed through agency. Research stress was associated with lower academic competence primarily through reduced agency and, to a lesser extent, through the sequential association involving agency and research role identity. The integrated model also showed a significant sequential indirect association from support through stress, agency, and identity to competence. These findings are consistent with a resource-based rather than deficit-based interpretation of doctoral stress and highlight the possible role of agentic engagement in developing scholarly competence from external resources.

## 1. Introduction

Doctoral students’ academic competence has received increasing research attention over the past two decades. It is widely recognized that cultivating robust academic competence, including methodological rigor and theoretical reasoning, is significantly related to doctoral training quality, professional socialization, and psychological well-being (Guo & Liu, 2026; Han et al., 2026; Levecque et al., 2017). To facilitate doctoral training, it is necessary to understand how academic competence develops in different high-stakes contexts, including contexts in which research pressure may be associated with lower scholarly competence (Y. T. Huang & Xu, 2020; Jarvis & Mok, 2019; Shin & Harman, 2009).

The well-established JD–R framework and recent literature suggest that doctoral academic outcomes are associated with job resources, such as supervisor support, and job demands, such as research stress (Bakker & Demerouti, 2024). Existing research has examined associations among external environments, student well-being, and attrition within the JD–R framework (Dericks et al., 2019; Levecque et al., 2017; Pyhältö et al., 2012), but less attention has been paid to academic competence as a developmental outcome. Few studies have examined doctoral students’ personal resources, specifically agency and research role identity, as possible internal pathways linking external conditions with scholarly capability (McAlpine & Amundsen, 2009; Sun & Trent, 2020).

Doctoral students at Chinese research universities are expected to produce quantifiable research outputs and often face substantial research pressure associated with the “Double First-Class” initiative, hierarchical supervisory relationships, and performance evaluation systems (Horta & Li, 2023; Moshtari & Schleper, 2025). Doctoral training is typically organized as a four-year program under a supervisor-responsibility system, in which each student is assigned a primary supervisor upon admission, and the supervisory relationship extends across the program, from coursework and topic selection in the early years to research collaboration and dissertation supervision later. This study therefore examines the associations of supervisor support and research stress with academic competence and tests a sequential indirect pathway involving agency and research role identity.

## 2. Literature Review and Hypothesis Development

### 2.1. The JD–R Framework in the Doctoral Context

The Job Demands–Resources (JD–R) model and its educational extension, Study Demands–Resources (SD–R), provide a framework for understanding how environmental conditions are associated with individual outcomes (Bakker & Demerouti, 2007; Bakker et al., 2023; Bakker & Mostert, 2024). The theory distinguishes a health-impairment pathway, in which sustained demands are associated with reduced energy, from a motivational pathway, in which resources are associated with engagement and growth (Bakker & Demerouti, 2024). While recent studies have applied JD–R to doctoral education, they have predominantly focused on well-being or attrition (Levecque et al., 2017; Pyhältö et al., 2012). Relatively few studies have examined academic competence, a developmental outcome relevant to future researchers. Traditional JD–R applications may also treat individuals as passive recipients of environmental stimuli. We therefore integrate personal resources, specifically doctoral student agency and research role identity, as possible internal pathways through which external conditions are associated with scholarly capability.

### 2.2. Support, Agency, Identity, and Academic Competence

Supervisor support is widely recognized as a job resource in doctoral training. In the hypothesized model, supervisor support is proposed to be positively associated with academic competence, with doctoral student agency specified as a possible intermediary pathway.

When supervisors provide guidance, encouragement, and resources, they may create a “secure base” that is expected to be associated with greater student ownership of research projects. Greater ownership is expected to be associated with higher doctoral student agency, including more proactive self-regulation and feedback seeking (Goller & Harteis, 2014).

The model specifies agency before identity as a theoretical ordering, not as an observed temporal sequence. Social cognitive perspectives suggest that professional identity may be enacted through repeated agentic behaviors. In the model, greater agency is expected to be associated with stronger research role identity as students engage in research tasks and become more familiar with the values and norms of the academic community (Chen, 2025; McAlpine et al., 2014; Sweitzer, 2009). Research role identity is, in turn, expected to be positively associated with academic competence, with deeper engagement in complex scholarly tasks specified as a possible pathway (McAlpine & Amundsen, 2009; Savva & Nygaard, 2021; Wilkin et al., 2023). Thus, we posit:

**Hypothesis** **1.** *Supervisor support has a significant total association with academic competence.*

**Hypothesis** **2.** *Supervisor support is indirectly associated with academic competence through doctoral student agency.*

**Hypothesis** **3.** *Supervisor support is indirectly associated with academic competence through research role identity.*

### 2.3. Research Stress and Academic Competence

Research stress in this study refers to perceived pressure associated with high publication standards and graduation requirements in the “publish or perish” culture (Guo & Liu, 2026; Horta & Li, 2023). We hypothesize that chronic research stress may be associated with fewer personal resources available for scholarly development. Conservation of Resources (COR) theory proposes that resource loss can initiate a loss cycle (Hobfoll, 2001), and prolonged stress may be associated with greater investment in coping with anxiety and uncertainty. Such demands may therefore be associated with less engagement in proactive agentic behaviors such as critical thinking and exploratory work (Stubb et al., 2011).

At the same time, research stress could be associated with fewer personal resources available for doctoral work. High academic demands may be associated with less proactive planning and autonomous decision-making (Sverdlik et al., 2018), and stress-related isolation has been linked to reduced relational agency, such as the willingness to seek feedback from supervisors (Hopwood, 2010). Lower agentic behavior may, in turn, be associated with weaker identity formation, as students may struggle to validate their self-concept as independent scholars (Castelló et al., 2021). The proposed “loss spiral” therefore describes a hypothesized pattern of associations: higher stress may be accompanied by lower agency, which may be associated with weaker identity development and, ultimately, lower academic competence. Thus, we propose:

**Hypothesis** **4.** *Research stress has a significant total association with academic competence.*

**Hypothesis** **5.** *Research stress is indirectly associated with academic competence through doctoral student agency.*

**Hypothesis** **6.** *Research stress is indirectly associated with academic competence through research role identity.*

### 2.4. Serial Indirect Associations: From “Doing” to “Being” and the Inverse Association with Stress

This study examines whether agency and identity form a sequential indirect association in the proposed model. While both are considered personal resources, the model specifies a sequence rather than parallel pathways. Theory on professional socialization suggests a “doing-to-being” trajectory: agency involves the enactment of research practices, whereas identity involves the internalization of the research role (McAlpine & Amundsen, 2009). Prior theory and longitudinal evidence suggest a possible sequential ordering between agency and identity (Mantai, 2019); the proposed model specifies this ordering as a theoretical sequence that cannot be tested temporally with the present cross-sectional design.

According to the JD–R model (Bakker & Demerouti, 2024), supervisor support may be associated with lower perceived stress and higher agency. We therefore specify a serial indirect pathway from supervisor support through research stress, agency, and identity to academic competence.

**Hypothesis** **7.** *Supervisor support is indirectly associated with academic competence through a sequential pathway involving agency and research role identity (Support → Agency → Identity → Competence).*

**Hypothesis** **8.** *Research stress is indirectly associated with academic competence through a sequential pathway involving agency and research role identity (Stress → Agency → Identity → Competence).*

**Figure 1:**
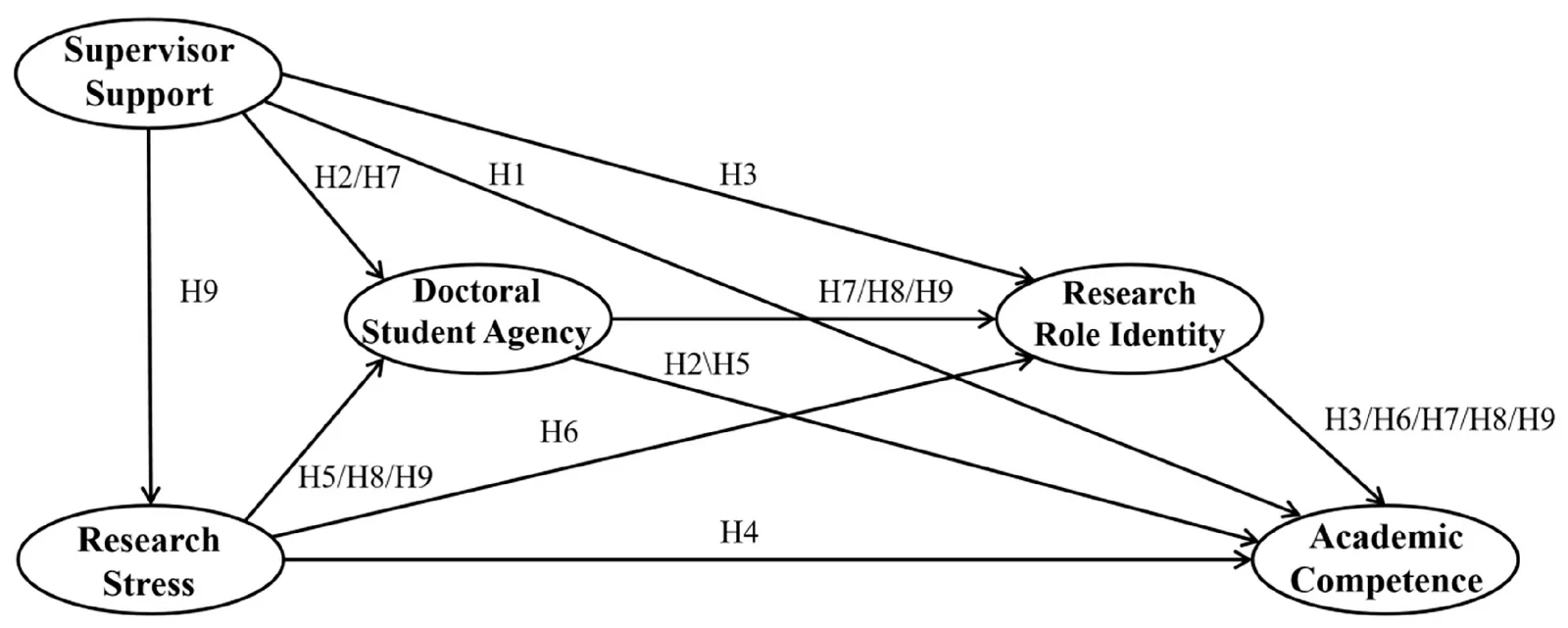
Conceptual model: the hypothesized dual-pathway model.

**Hypothesis** **9.** *Supervisor support will be indirectly associated with academic competence through a sequential pathway involving research stress, agency, and research role identity (Support → Stress → Agency → Identity → Competence). The hypothesized model is depicted in Figure 1.*

## 3. Methodology

### 3.1. Participants and Procedure

To address the research questions, a cross-sectional survey was conducted with full-time doctoral students at three comprehensive universities in Beijing, China. A purposive convenience sample was used because these institutions offered access to doctoral students across disciplines and years of study and operate under the same national doctoral education policy framework. The universities are not identified individually to protect institutional anonymity. This sample is not claimed to represent the participating universities, Beijing, or doctoral students nationally. Participants were recruited through Wenjuanxing, and the survey link was distributed through WeChat. Participation was voluntary and anonymous. No formal quota targets or sampling frame were available. Recruitment prioritized variation in year of study and sought to include students from different disciplines and genders; “balance sought” therefore refers to deliberate coverage during recruitment rather than formal proportional quotas. The questionnaire was distributed in September 2025. In total, 469 doctoral students completed the questionnaire after providing informed consent. After screening for completeness and removing 33 incomplete or invalid responses, 436 valid questionnaires were retained (usable-response proportion = 93.0%, 436/469). Ethical approval was obtained from the Ethics Committee of the Faculty of Education at Beijing Normal University (approval no. BNU202506100035; approval date: 17 July 2025). The present study is a secondary analysis of the dataset used in the DSAS scale-development study (L. Huang et al., 2025); whereas that study focused on scale development and psychometric validation, the present study examines a substantive sequential indirect model.

Among the participants, 64.0% were female, and 36.0% were male. Academically, 69.0% of the participants were from humanities and social sciences, while 31.0% were from Science, Technology, Engineering, Mathematics (STEM) fields. Notably, 77.3% were enrolled in disciplines recognized under China’s “Double First-Class” initiative, reflecting the high-stakes research environment targeted by this study. Their detailed demographic characteristics are shown in Table 1.

### 3.2. Measures

All instruments were administered in Chinese. The academic competence, research stress, research role identity, and doctoral student agency measures were either originally developed in Chinese or adapted for Chinese doctoral students; the supervisor support measure used an established Chinese adaptation of Overall et al. (2011), as reported by Zhu (2019). No new translation or back-translation was undertaken. Item content had been reviewed during the original scale development or adaptation process. The final questionnaire contained 63 items scored on a 5-point Likert scale ranging from 1 (strongly disagree) to 5 (strongly agree). Confirmatory Factor Analysis (CFA) was used to examine measurement structure and validity, and internal consistency was assessed with Cronbach’s alpha. Discriminant validity was assessed using the Fornell–Larcker criterion (Fornell & Larcker, 1981). Table 2 reports fully standardized factor loadings. These measures have also been used in prior research with Chinese graduate students: the supervisor-support adaptation has been used in several Chinese supervision studies (Y. Wang et al., 2016; B. Hu, 2017; Zhu, 2019; Zhao & Jiang, 2020), and the academic-competence and research-stress measures have been applied in Chinese graduate-education research (Chi, 2017; Y. Zhang et al., 2013). These applications have examined research output, innovation behavior, creativity, academic adaptation, and graduate-education outcomes, providing prior evidence for the appropriateness of the measures with Chinese graduate populations. Detailed item mapping, deletion rationale, and final psychometric evidence are provided in Supplementary Tables S1–S6.

Academic Competence: Drawing on Chi’s (2017) conceptualization and scale items, an 18-item measure covering three dimensions was developed for the doctoral context. Minor wording adjustments were made for the doctoral research context while retaining the construct definitions and substantive content of the source items. Four items were removed because of cross-loadings or near-equal loadings, yielding 14 final items. The final 14-item measure showed α = 0.93 in the present sample, and CFA supported its three-dimensional structure. The present measurement model is reported in Table 2.

Supervisor Support: The measure was based on Overall et al. (2011) and the Chinese adaptation reported by Zhu (2019). The wording was adapted for doctoral supervision, and a resource-support dimension was added based on interview-derived content and prior Chinese supervision research. The resource-support items concern research funding, participation in research projects, and help with manuscript submission. Four items were removed through sequential model refinement using CFA modification indices, theoretical consistency, a minimum of three items per dimension, and the avoidance of cross-dimension residual correlations. The final measure contained 12 items across four dimensions, with three items per dimension. The final measure showed α = 0.94 in the present full sample, and CFA supported the four-dimensional structure.

Research Stress: Five items were adapted from Y. Zhang et al. (2013) to capture perceived pressure related to research tasks and degree-related evaluation. The item stems were contextualized for doctoral research while preserving the original single-factor conceptualization. One item showed high residual covariances with multiple items and was removed, yielding four final items. The final four-item model showed acceptable fit, and α was 0.90 in the present full sample. A sample item is “External research evaluation standards place considerable pressure on me.”

Doctoral Student Agency: The DSAS (L. Huang et al., 2025), developed and validated in the published companion study using the same dataset, contains 27 items across seven first-order dimensions and three second-order dimensions. The initial item pool was generated from interviews and qualitative coding and refined through structured review and item analysis to produce the final 27-item measure. The DSAS was used to measure students’ intentional and proactive actions during professional socialization. A sample item is “I will adjust my communication style according to my supervisor’s guidance style to facilitate the resolution of the problem.” The internal consistency in the present sample was high (α = 0.94). Because the companion study and this study share the same dataset, the higher-order structure is treated as a pre-specified measurement model rather than independently validated here.

Research Role Identity: The six-item measure was compiled for this study from prior research on researcher identity and related constructs (Fu, 2020; Li, 2020; Perez et al., 2014; J. Zhang, 2014); it was not adopted verbatim from a single source. The wording was revised to use “researcher” and “scientific worker” as the referent roles, and the items cover recognition as a researcher, the importance of the researcher role, pride, group belonging, self-concept, and responsibility. The final six-item measure showed high reliability in the present sample (α = 0.94), and CFA supported a one-factor structure.

### 3.3. Statistical Analysis

Before testing the hypothesized model, common method bias (CMB) was assessed using Harman’s single-factor test. An exploratory factor analysis including all study items revealed 12 factors with eigenvalues greater than 1, and the first factor accounted for 32.93% of the total variance, below the recommended 40% threshold (Podsakoff et al., 2003). The survey was administered anonymously, and the scales for different constructs were presented in separate sections as procedural remedies.

CFA and Structural Equation Modeling (SEM) were conducted in IBM SPSS Amos 26.0, with descriptive statistics computed in SPSS 25.0; parameters were estimated using maximum likelihood. Because the 33 incomplete or invalid responses were excluded before analysis, the final sample contained no missing values for the analyzed variables. Model fit was evaluated using the χ^2^/df ratio, CFI, TLI, RMSEA, and SRMR; adequate fit was indicated by χ^2^/df < 5, CFI and TLI ≥ 0.90, and RMSEA ≤ 0.08 (L. T. Hu & Bentler, 1999). The model was over-identified (df = 242), and all reported path coefficients are fully standardized. Owing to the number of items in the multidimensional measures, theoretically derived domain scores were used as indicators for agency, supervisor support, and academic competence to reduce model complexity and improve parameter stability (Little et al., 2002). Doctoral student agency was specified as a higher-order latent construct indicated by its seven domain scores, following the structure reported in L. Huang et al. (2025). Supervisor support was indicated by four domain scores, academic competence by three domain scores, and research stress and research role identity by their individual items.

## 4. Results

### 4.1. Descriptive Statistics and Correlations

Table 3 presents the means, standard deviations, and correlation coefficients for all variables. On the 5-point scales, the mean scores were as follows: supervisor support (M = 3.67, SD = 0.80), research role identity (M = 3.62, SD = 0.80), academic competence (M = 3.58, SD = 0.56), doctoral student agency (M = 3.57, SD = 0.56), and research stress (M = 3.58, SD = 0.90). As shown in Table 3, supervisor support, doctoral student agency, research role identity, and academic competence were significantly and positively intercorrelated (all *p* values < 0.001). Conversely, research stress was negatively correlated with these variables (all *p* values < 0.001). These associations provide preliminary evidence consistent with the hypothesized motivational and depletion pathways.

### 4.2. Direct and Total Associations of Support and Stress

The study first evaluated the structural model to test the direct and total associations of supervisor support and research stress with academic competence. The model fitted the data well: χ^2^(242) = 769.946, *p* < 0.001, χ^2^/df = 3.18, CFI = 0.93, TLI = 0.92, RMSEA = 0.07, 90% CI [0.065, 0.076], and SRMR = 0.06. Amos 26.0 does not directly report SRMR for this model. SRMR was therefore computed from the standardized differences between the sample and model-implied covariance matrices (using 24 indicators), yielding 0.059, which rounds to 0.06; the computation is documented in Supplementary Note S1. Because the structural model was saturated at the latent level, the five-factor measurement model and the structural model were statistically equivalent and yielded identical fit. Supervisor support had a significant positive total association with academic competence (β = 0.44, *p* < 0.001; H1), and research stress had a significant negative total association with academic competence (β = −0.22, *p* < 0.001; H4). With the mediators included, the direct paths from supervisor support (β = −0.06, *p* > 0.05) and research stress (β = 0.00, *p* > 0.05) to academic competence were not significant. Together with the significant indirect associations reported below, these results indicate that no significant direct associations were observed, whereas the hypothesized indirect associations were significant.

### 4.3. Estimated Indirect Associations Through Doctoral Student Agency and Research Role Identity

To examine the specified indirect pathways (Hypotheses 2, 3, and 5–9), bias-corrected bootstrapping with 2000 resamples was employed. The estimateddirect, total, and indirect associations are summarized in Table 4.

Regarding the motivational pathway, the total indirect association between supervisor support and academic competence was significant (β = 0.50, 95% CI [0.42, 0.60]). Specifically, the indirect association via agency alone was significant (β = 0.35, 95% CI [0.25, 0.45]), supporting Hypothesis 2. The indirect association via research role identity alone was also significant (β = 0.04, 95% CI [0.01, 0.08]), supporting Hypothesis 3.

Regarding the stress pathway, the total indirect association between research stress and academic competence was significant (β = −0.22, 95% CI [−0.31, −0.13]). The specific indirect association via agency alone was significant and negative (β = −0.19, 95% CI [−0.27, −0.11]), supporting Hypothesis 5. However, the indirect pathway via research role identity alone was not significant (β = 0.001, 95% CI [−0.02, 0.02], *p* > 0.05), failing to support Hypothesis 6.

**Figure 2:**
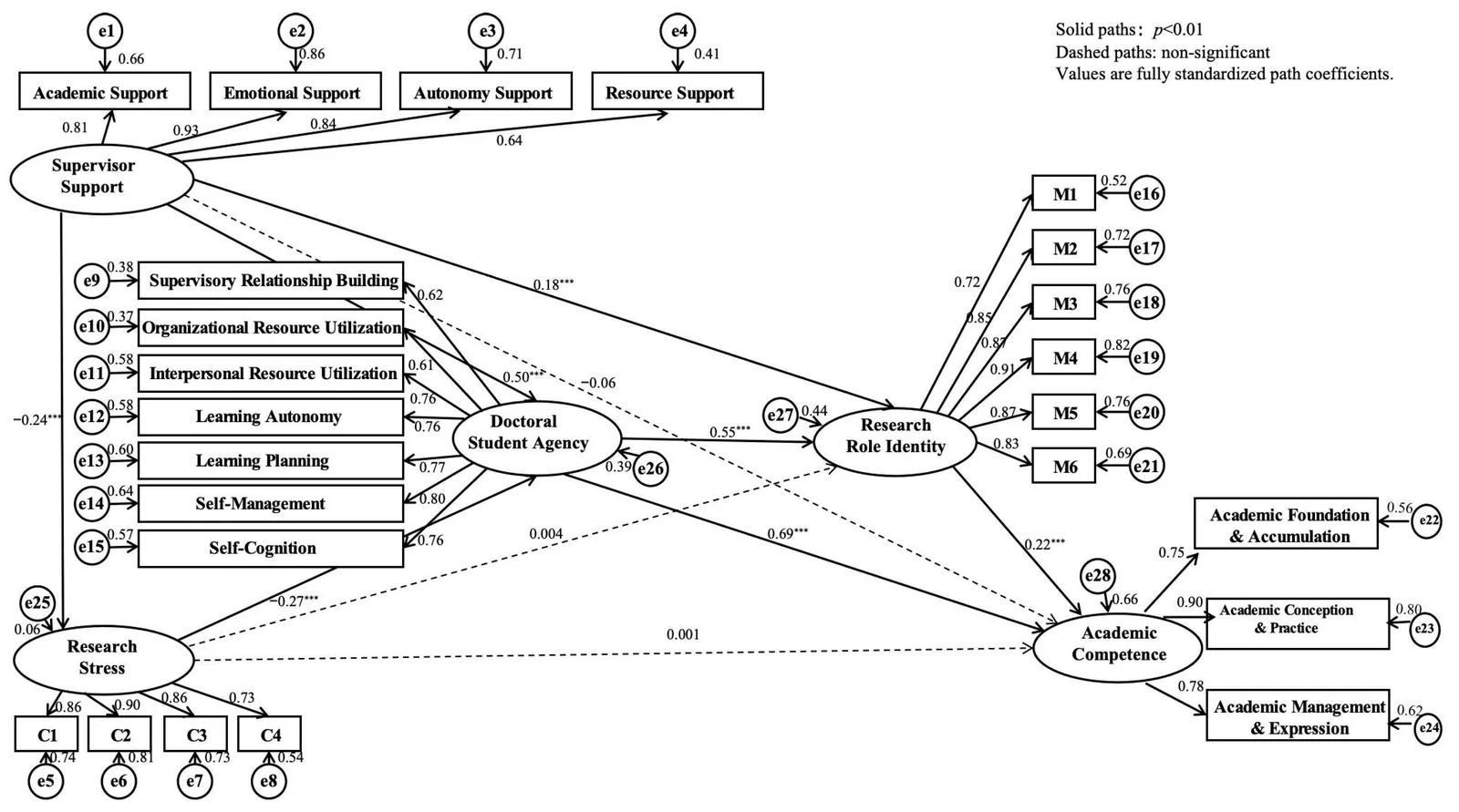
Structural model. Note. The path coefficients from Research Stress to Academic Competence and Research Role Identity are reported to three decimal places (0.001 and 0.004, respectively) to provide greater precision for estimates close to zero; all other coefficients are reported to two decimal places. *** *p* < 0.001.

The sequential indirect associations were then examined. The pathway from supervisor support to academic competence through agency and then research role identity was significant (β = 0.06, 95% CI [0.03, 0.11]), supporting Hypothesis 7. The corresponding pathway from research stress through agency and identity was also significant (β = −0.03, 95% CI [−0.06, −0.02]), supporting Hypothesis 8. In the integrated model, the specified sequence from supervisor support through research stress, agency, and identity to competence was significant (β = 0.01, 95% CI [0.003, 0.02]), supporting Hypothesis 9. This finding is consistent with an inverse association between supervisor support and perceived stress that forms part of an indirect association with competence. A complementary pathway from supervisor support through research stress and agency to competence was also significant (β = 0.05, 95% CI [0.02, 0.08]). Figure 2 presents the standardized coefficients.

## 5. Discussion

### 5.1. Summary of Key Findings

Based on a sample of doctoral students in three comprehensive universities in Beijing, China, this study used the JD–R framework to examine the associations linking environmental conditions with academic competence. Academic competence was expected to be associated with both external factors (e.g., supervisor support) and internal factors (e.g., research engagement and agency) (J. Wang, 2025). The observed pattern was consistent with a dual-pathway model: supervisor support was associated with academic competence through agency and research role identity, whereas research stress was associated with lower competence through lower agency.

### 5.2. The Role of Agency in the Observed Associations

While extensive research has examined the supervisor–student relationship, less work has examined the internal variables that may account for its association with doctoral outcomes (Sverdlik et al., 2018). In this study, supervisor support was associated with academic competence through personal resources, and the largest estimated specific indirect association was observed through agency. This pattern is more consistent with a resource-oriented interpretation than with a simple transfer model in which competence is transmitted directly from supervisor to student.

### 5.3. The “Doing-to-Being” Trajectory: A Sequential Indirect Association

The significant sequential pathway from supervisor support through agency and identity to competence is consistent with theoretical accounts of professional socialization in which agentic action is associated with identity construction. This finding also aligns with a possible “doing-to-being” trajectory (McAlpine & Amundsen, 2009). In this interpretation, agency represents the behavioral component of research engagement, whereas research role identity represents the internalization of the researcher role.

### 5.4. Supervisor Support and Perceived Research Stress

Research stress was associated with lower academic competence primarily through lower doctoral student agency. This pattern aligns with the “loss spiral” interpretation in Conservation of Resources theory (Hobfoll, 2001). In China, institutional publication and graduation requirements have been linked to research stress among doctoral students (Horta & Li, 2023). The integrated model also showed a small but significant indirect association from supervisor support through lower perceived stress, agency, and identity to competence (β = 0.01). This pattern is consistent with the idea that supportive relationships may accompany lower perceived stress and, indirectly, higher competence.

### 5.5. Theoretical Implications

This study contributes to the existing literature in three ways. First, it applies the JD–R model to doctoral education by examining academic competence as a developmental outcome distinct from publication-based performance metrics. Second, it describes the indirect associations observed through personal resources. The largest estimated specific indirect association was observed through agency, highlighting the relevance of personal resources to doctoral development. Third, the observed ordering is consistent with a sequential association between agency and identity, rather than establishing a developmental sequence.

### 5.6. Practical Implications

Because the design is cross-sectional, these practice implications describe directions suggested by the observed association pattern rather than demonstrated intervention effects. For supervisors, the pattern is consistent with the possibility that support may be most useful when it also creates opportunities for student autonomy, goal setting, and decision-making. For institutions, the negative association between research stress and agency indicates that very high pressure may be counterproductive, but the present design cannot establish that reducing pressure would improve agency. Institutional supports such as mentoring and stress-management resources may therefore merit consideration. For doctoral students, the findings are consistent with the potential value of active engagement, such as seeking feedback, building networks, and managing time.

## 6. Limitations and Future Directions

Several limitations of this study should be noted. First, the cross-sectional design precludes causal inferences and cannot establish the temporal ordering of agency and identity; the reverse or reciprocal ordering (e.g., identity → agency) remains plausible and was not formally tested. In addition, no separate expert panel, pilot study, or cognitive interview was conducted for the four non-DSAS measures in this study; content validity for these measures therefore rests on prior validation, conceptual alignment, and the current construct-validity evidence. Future longitudinal or experience-sampling studies are needed to adjudicate competing orderings. Second, all variables were assessed through self-report questionnaires collected at a single time point from a single source, which may introduce common-method variance and subjective bias. Future work should incorporate objective performance indicators or supervisor ratings and consider multi-source or multi-wave designs. Third, the sample was recruited through an online, non-probability approach at three comprehensive universities in Beijing, China; no sampling frame or number of invited eligible participants was available, so a conventional response rate could not be calculated. These features limit generalizability. Fourth, the DSAS was developed using the same dataset; therefore, the higher-order measurement structure was not independently validated in the present sample. Fifth, the reported direct, indirect, and total estimates are model-derived associations rather than causally identified effects. Future models could also incorporate peer support and departmental climate to provide a broader ecological view of doctoral development.

## 7. Conclusions

This study developed and examined a dual-pathway model of doctoral students’ academic competence within the JD–R framework. Based on 436 doctoral students from three comprehensive universities in Beijing, China, supervisor support and research stress were not directly associated with academic competence when agency and research role identity were included in the model. The largest estimated specific indirect association was observed through agency, and the sequential “doing-to-being” association was significant. These findings are consistent with the interpretation that doctoral competence is associated with students’ agentic engagement and internalized research identity.

## Figures and Tables

**Table 1:** Distribution of survey sample (N = 436).

Variable	Category	Frequency (N)	Percentage (%)
Year of Study	Year 1	99	22.71
	Year 2	71	16.28
	Year 3	83	19.04
	Year 4	120	27.52
	Year 5 and above	63	14.46
Gender	Male	157	36
	Female	279	64
Discipline	STEM	135	30.96
	Humanities & Social Sciences	301	69.04
Discipline Level	Double First-Class Discipline	337	77.29
	Non-Double First-Class	99	22.71
Admission Type	General Admission/Application–Assessment	371	85.09
	Special Admission (e.g., Direct PhD)	65	14.91
Work Experience	Research-related experience	78	17.89
	Non-research experience	83	19.04
	No work experience	275	63.07

“Double First-Class” refers to the Chinese government’s higher education development initiative.

**Table 2:** Reliability and convergent validity analyses.

Construct	Dimensions	No. of Items	Factor Loadings	Cronbach’s α	CR	AVE	Source
Academic Competence	Total Scale	14	[0.75, 0.90]	0.93	0.85	0.66	Chi (2017), adapted
	1. Academic Conception & Practice	[7]	[0.60, 0.84]				
	2. Academic Expression & Management	[4]	[0.67, 0.74]				
	3. Academic Foundation & Accumulation	[3]	[0.78, 0.88]				
Supervisor Support	Total Scale	12	[0.64, 0.93]	0.94	0.89	0.66	Overall et al. (2011); Zhu (2019), adapted
	1. Academic Support	[3]	[0.81, 0.91]				
	2. Emotional Support	[3]	[0.88, 0.92]				
	3. Autonomy Support	[3]	[0.66, 0.95]				
	4. Resource Support	[3]	[0.75, 0.80]				
Research Stress	Unidimensional	4	[0.73, 0.90]	0.90	0.91	0.71	Y. Zhang et al. (2013), revised
Doctoral Student Agency	Total Scale	27	[0.60, 0.81]	0.94	0.89	0.53	L. Huang et al. (2025)
	1. Self-Cognition	[3]	[0.76, 0.78]				
	2. Self-Management	[4]	[0.64, 0.76]				
	3. Academic Planning	[5]	[0.62, 0.83]				
	4. Academic Autonomy	[4]	[0.73, 0.84]				
	5. Organizational Resource Utilization	[3]	[0.64, 0.78]				
	6. Interpersonal Resource Utilization	[5]	[0.64, 0.78]				
	7. Student-Supervisor Relationship Construction	[3]	[0.73, 0.82]				
Research Role Identity	Unidimensional	6	[0.70, 0.93]	0.94	0.94	0.71	Multiple sources (see Section 3.2)

Note: Factor loadings for each dimension are standardized item-level loadings from the confirmatory factor analysis of that construct. For multi-dimensional constructs, the loading range reported for the total scale refers to the standardized loadings of the dimension (domain) scores on the latent factor in the full structural model.

**Table 3:** Descriptive statistics, correlations, and discriminant validity.

Variable	M	SD	1	2	3	4	5
1. Supervisor Support	3.67	0.80	0.81				
2. Research Stress	3.58	0.90	−0.20 ***	0.84			
3. Doctoral Student Agency	3.57	0.56	0.50 ***	−0.35 ***	0.73		
4. Research Role Identity	3.62	0.80	0.44 ***	−0.24 ***	0.59 ***	0.84	
5. Academic Competence	3.58	0.56	0.39 ***	−0.28 ***	0.70 ***	0.60 ***	0.81

Note: N = 436. The bold diagonal elements are the square roots of the average variance extracted (AVE) from the measurement model reported in Table 2; for discriminant validity, each square root of AVE should exceed the correlations in its row and column (Fornell & Larcker, 1981). Off-diagonal elements are Pearson correlation coefficients. *** *p* < 0.001.

**Table 4:** Summary of estimated direct and indirect associations.

Pathway	Estimate (β)	SE	95% CI Lower	95% CI Upper	Result
1. Motivational Path (Supervisor Support → Academic Competence)					
H1: Supervisor Support → Academic Competence (Total Association)	0.44 ***	0.05	0.33	0.53	Supported
Direct Association: Supervisor Support → Academic Competence	−0.06	0.06	−0.17	0.05	Not significant
H2 (Specific Indirect Association): Supervisor Support → Doctoral Student Agency → Academic Competence	0.35 ***	0.05	0.25	0.45	Supported
H3 (Specific Indirect Association): Supervisor Support → Research Role Identity → Academic Competence	0.04 **	0.02	0.01	0.08	Supported
2. Stress Pathway (Research Stress → Academic Competence)					
H4: Research Stress → Academic Competence (Total Association)	−0.22 ***	0.05	−0.32	−0.11	Supported
Direct Association: Research Stress → Academic Competence	0.00	0.05	−0.09	0.09	Not significant
H5 (Specific Indirect Association): Research Stress → Doctoral Student Agency → Academic Competence	−0.19 ***	0.04	−0.27	−0.11	Supported
H6 (Specific Indirect Association): Research Stress → Research Role Identity → Academic Competence	0.001	0.01	−0.02	0.02	Not Supported
3. Serial Indirect Associations					
H7 (Specific Indirect Association): Supervisor Support → Doctoral Student Agency → Research Role Identity → Academic Competence	0.06 ***	0.02	0.03	0.11	Supported
H8 (Specific Indirect Association): Research Stress → Doctoral Student Agency → Research Role Identity → Academic Competence	−0.03 ***	0.01	−0.06	−0.02	Supported
H9 (Specific Indirect Association): Supervisor Support → Research Stress → Doctoral Student Agency → Research Role Identity → Academic Competence	0.01 ***	0.003	0.003	0.02	Supported
Complementary Specific Indirect Association: Supervisor Support → Research Stress → Doctoral Student Agency → Academic Competence	0.05 ***	0.01	0.02	0.08	Significant
4. Total Indirect Association					
Total Indirect Association: Supervisor Support → Academic Competence	0.50 ***	0.05	0.42	0.60	Significant
Total Indirect Association: Research Stress → Academic Competence	−0.22 ***	0.04	−0.31	−0.13	Significant

Note: β = fully standardized coefficient; SE = bootstrap standard error; CI = bias-corrected bootstrap confidence interval. Bootstrap estimates were based on 2000 resamples. Total associations (H1 and H4) equal the direct associations plus the total indirect associations within rounding. Rows labeled as specific indirect associations report individual model-implied pathways; these estimates may not sum exactly to the total indirect association because of rounding. Estimates are model-derived associations from a cross-sectional model rather than causally identified effects. ** *p* < 0.01, *** *p* < 0.001.

## Data Availability

The datasets generated during and/or analyzed during the current study are available from the first author upon reasonable request.

## Supplementary Materials

The following supporting information can be downloaded at https://www.mdpi.com/article/10.3390/bs16101941/s1, Table S1: Academic Competence: Item Mapping and Refinement; Table S2: Supervisor Support: Item Mapping and Refinement; Table S3: Research Stress: Item Mapping and Refinement; Table S4: Research Role Identity: Item Mapping; Table S5: Doctoral Student Agency Scale: Validated Structure; Table S6: Final Psychometric Evidence for the Revised Measures; Supplementary Note S1 documents the SRMR calculation. Sources cited in these supplementary tables are cited in the main text and listed in the main reference list: Chi (2017); Overall et al. (2011); Zhu (2019); Y. Zhang et al. (2013); J. Zhang (2014); Fu (2020); Li (2020); Perez et al. (2014); and L. Huang et al. (2025).
