# Supplementary material for: From “Doing” to “Being”: How Agency and Identity Mediate the Associations of Support and Stress with Doctoral Competence in Chinese Universities
Source: Behav Sci (Basel). 2026 Oct 19;16(10):1941. doi: 10.3390/bs16101941 (PMC13701336; doi:10.3390/bs16101941)
Supplement: Supplementary file 1 [file behavsci-16-01941-s001.zip › behavsci-4570240-supplementary.pdf]

Supplementary Material

Manuscript: From “Doing” to “Being”: How Agency and Identity Mediate the Associations of Support and Stress with Doctoral Competence in Chinese Universities

Note. The item descriptions below are concise summaries rather than verbatim reproductions of copyrighted instrument items. They are provided to make the item numbering, dimension assignment, and deletion decisions verifiable without replacing the original instruments. All sources cited in this supplement are also cited in the main text and listed in the main reference list: Chi (2017); Overall et al. (2011); Zhu (2019); Zhang et al. (2013); Zhang (2014); Fu (2020); Li (2020); Perez et al. (2014); and Huang et al. (2025).

Table S1. Academic Competence: Item Mapping and Refinement

Item ID	Intended dimension	Brief item content	Status	Final assignment	Decision basis
AC01	Academic foundation and accumulation	Knowledge base and theoretical system of the discipline	Retained	Final three-item foundation dimension	Retained in final model
AC02	Academic foundation and accumulation	Frontier knowledge in the discipline	Retained	Final three-item foundation dimension	Retained in final model
AC03	Academic foundation and accumulation	Methodology and basic methods or skills	Retained	Final three-item foundation dimension	Retained in final model
AC04	Academic conception and practice	Obtains research literature through multiple sources	Retained	Final seven-item conception/practice dimension	Retained in final model
AC05	Academic conception and practice	Analyzes, synthesizes, and summarizes literature	Retained	Final seven-item conception/practice dimension	Retained in final model
AC06	Academic conception and practice	Identifies key unresolved research problems	Retained	Final seven-item conception/practice dimension	Retained in final model
AC07	Academic conception and practice	Develops workable solutions to research problems	Retained	Final seven-item conception/practice dimension	Retained in final model
AC08	Academic conception and practice	Constructs a clear research logic and framework	Retained	Final seven-item conception/practice dimension	Retained in final model
AC09	Academic conception and practice	Uses statistical results to identify substantive issues	Retained	Final seven-item conception/practice dimension	Retained in final model
AC10	Academic conception and practice	Draws logically valid conclusions through analysis	Retained	Final seven-item conception/practice dimension	Retained in final model
AC11	Academic conception and practice	Evaluates strengths and limitations of prior research	Removed	Cross-loading or near-equal loadings across dimensions	Removed after cross-loading or near-equal loadings
AC12	Academic conception and practice	Generates distinctive insights when addressing research problems	Removed	Cross-loading or near-equal loadings across dimensions	Removed after cross-loading or near-equal loadings
AC13	Academic conception and practice	Improves or extends prior research	Removed	Cross-loading or near-equal loadings across dimensions	Removed after cross-loading or near-equal loadings
AC14	Academic expression and management	Communicates academic ideas in different settings	Retained	Final four-item expression/management dimension	Retained in final model
AC15	Academic expression and management	Applies academic writing conventions and communicates clearly	Retained	Final four-item expression/management dimension	Retained in final model
AC16	Academic expression and management	Capacity to lead a research project	Retained	Final four-item expression/management dimension	Retained in final model
AC17	Academic expression and management	Collaborates effectively in research teams	Retained	Final four-item expression/management dimension	Retained in final model
AC18	Academic expression and management	Follows research ethics and academic norms	Removed	Cross-loading or near-equal loadings across dimensions	Removed after cross-loading or near-equal loadings

Source: Chi (2017), adapted wording for the doctoral research context. Original 18 items; final 14 items. Full item wording is available from the source instrument, subject to copyright permissions.

Table S2. Supervisor Support: Item Mapping and Refinement

Item ID	Intended dimension	Brief item content	Status	Final assignment	Decision basis
SS01	Academic support	Guides literature and research-material searching	Removed	Model refinement	Removed during sequential CFA refinement
SS02	Academic support	Transmits research skills and experience	Retained	Academic support	Retained in final model
SS03	Academic support	Provides timely feedback on research outputs	Retained	Academic support	Retained in final model
SS04	Academic support	Discusses difficulties and gives useful advice	Retained	Academic support	Retained in final model
SS05	Emotional support	Shows warmth and concern when discussing research	Removed	Model refinement	Removed during sequential CFA refinement
SS06	Emotional support	Appreciates the student and supports positive academic self-perception	Retained	Emotional support	Retained in final model
SS07	Emotional support	Notices low mood and provides reassurance	Retained	Emotional support	Retained in final model
SS08	Emotional support	Encourages confidence in completing the dissertation	Retained	Emotional support	Retained in final model
SS09	Autonomy support	Values and respects the student's ideas and concerns	Retained	Autonomy support	Retained in final model
SS10	Autonomy support	Provides discretion in research decisions	Retained	Autonomy support	Retained in final model
SS11	Autonomy support	Encourages independent research work	Retained	Autonomy support	Retained in final model
SS12	Autonomy support (reverse worded)	Does not consider the student's wishes when setting research direction	Removed	Model refinement	Removed during sequential CFA refinement
SS13	Resource support	Introduces helpful academic contacts	Removed	Model refinement	Removed during sequential CFA refinement
SS14	Resource support	Provides research funding support	Retained	Resource support	Retained in final model
SS15	Resource support	Provides opportunities to participate in research projects	Retained	Resource support	Retained in final model
SS16	Resource support	Provides help with journal submission	Retained	Resource support	Retained in final model

Source: Overall et al. (2011), with the Chinese adaptation reported by Zhu (2019) and an interview-derived resource-support dimension. Original 16 items; final 12 items, with three items in each dimension.

Table S3. Research Stress: Item Mapping and Refinement

Item ID	Intended dimension	Brief item content	Status	Final assignment	Decision basis
RS01	Research stress	Worries frequently about completing research tasks	Retained	Final unidimensional scale	Retained in final model
RS02	Research stress	Feels considerable pressure from research work	Retained	Final unidimensional scale	Retained in final model
RS03	Research stress	Feels depressed or unhappy because of research work	Retained	Final unidimensional scale	Retained in final model
RS04	Research stress	External research evaluation standards create pressure	Retained	Final unidimensional scale	Retained in final model
RS05	Research stress	Pressure from sustained time investment without good results	Removed	High residual covariance with multiple items	Removed after improvement in residual structure

Source: Zhang et al. (2013), contextualized for doctoral research tasks and degree-related evaluation. Original five items; final four-item unidimensional scale.

Table S4. Research Role Identity: Item Mapping

Item ID	Intended dimension	Brief item content	Status	Final assignment	Decision basis
RI01	Research role identity	Sees oneself as a researcher or scientific worker	Retained	Final unidimensional scale	Retained in final model
RI02	Research role identity	Regards the researcher role as important for personal development	Retained	Final unidimensional scale	Retained in final model
RI03	Research role identity	Feels proud of being a researcher or scientific worker	Retained	Final unidimensional scale	Retained in final model
RI04	Research role identity	Feels a strong sense of belonging to the researcher community	Retained	Final unidimensional scale	Retained in final model
RI05	Research role identity	Regards the researcher identity as an important part of self-image	Retained	Final unidimensional scale	Retained in final model
RI06	Research role identity	Feels responsibility and a sense of mission as a researcher	Retained	Final unidimensional scale	Retained in final model

Source: compiled from Zhang (2014), Fu (2020), Li (2020), and Perez et al. (2014), with wording revised for the doctoral researcher role. Six items retained.

Table S5. Doctoral Student Agency Scale: Validated Structure

First-order dimension	Second-order dimension	Items	Source
Self-cognition	Self-agency	3	Huang et al. (2025)
Self-management	Self-agency	4	Huang et al. (2025)
Academic planning	Learning agency	5	Huang et al. (2025)
Academic autonomy	Learning agency	4	Huang et al. (2025)
Organizational resource utilization	Resource agency	3	Huang et al. (2025)
Interpersonal resource utilization	Resource agency	5	Huang et al. (2025)
Student-supervisor relationship construction	Resource agency	3	Huang et al. (2025)

The DSAS was developed and validated in the published companion study using the same dataset. The present article treats its higher-order structure as a pre-specified measurement model and does not claim independent validation in this sample.

Table S6. Final Psychometric Evidence for the Revised Measures

Measure	Final structure	Final reliability	Final validity evidence	Source
Academic competence	14 items; three dimensions	Present-sample α = .93	CFA supported the three-dimensional structure; dimension loading ranges [.60-.84], [.67-.74], and [.78-.88]; CR = .85; AVE = .66	Chi (2017), adapted
Supervisor support	12 items; four dimensions (3 items each)	Present-sample α = .94	CFA supported the four-dimensional structure; dimension loading ranges [.81-.91], [.88-.92], [.66-.95], and [.75-.80]; CR = .89; AVE = .66	Overall et al. (2011); Zhu (2019), adapted
Research stress	4 items; unidimensional	Present-sample α = .90	CFA supported the final unidimensional structure; item loadings [.73-.90]; CR = .91; AVE = .71	Zhang et al. (2013), adapted
Research role identity	6 items; unidimensional	Present-sample α = .94	CFA supported the one-factor structure; item loadings [.70-.93]; CR = .94; AVE = .71	Multiple sources
Doctoral Student Agency Scale	27 items; seven first-order and three second-order dimensions	Present-sample α = .94; published validation α = .941	Published validation supports the seven-factor and higher-order structure; the present study treats it as pre-specified because the datasets overlap	Huang et al. (2025)

Note S1. SRMR Calculation

Amos 26.0 did not directly report SRMR for the specified structural model. SRMR was therefore computed from the standardized lower-triangular residuals of the sample and model-implied covariance matrices, including the diagonal:

$SRMR = \text{sqrt}\{ \Sigma[(s_{ij} - \sigma_{ij}) / \text{sqrt}(s_{ii} \times s_{jj})]^2 / [p(p + 1) / 2] \}$, where p = 24 indicators. The computed value was .059, which rounds to .06.

Content-validity note. The supplementary material reports the final revised measures and their post-revision psychometric evidence. It does not reproduce the full development-stage validation history. Item-level mapping documents which items were retained or removed and why. No separate expert panel, pilot study, or cognitive interview was conducted for the four non-DSAS measures in this study; content validity for these measures therefore rests on source alignment, conceptual coverage, and the final construct-validity evidence.
